# Lymph node dissection for Siewert II esophagogastric junction adenocarcinoma: Erratum

**DOI:** 10.1097/MD.0000000000007316

**Published:** 2017-06-16

In the article, “Lymph node dissection for Siewert II esophagogastric junction adenocarcinoma”,^[1]^ which appeared in Volume 96, Issue 7 of *Medicine*, the authors’ affiliation should have appeared as “Department of Esophageal Cancer, Tianjin Medical University Cancer Institute and Hospital, National Clinical Research Center for Cancer, Key Laboratory of Cancer Prevention and Therapy of Tianjin City, Clinical Research Center for Cancer of Tianjin City, Tianjin, China.”
